# Prenatal diagnosis of *PLP1* duplication by single nucleotide polymorphism array in a family with Pelizaeus-Merzbacher disease

**DOI:** 10.18632/aging.202477

**Published:** 2021-01-11

**Authors:** Huili Xue, Aili Yu, Xuemei Chen, Na Lin, Min Lin, Hailong Huang, Liangpu Xu

**Affiliations:** 1 Fujian Key Laboratory for Prenatal Diagnosis and Birth Defect, Fujian Maternity and Child Health Hospital, Affiliated Hospital of Fujian Medical University, Gulou, Fuzhou 350001, Fujian Province, China; 2 Reproductive Medicine Center, Fujian Maternity and Child Health Hospital, Affiliated Hospital of Fujian Medical University, Gulou, Fuzhou 350001, Fujian Province, China

**Keywords:** single nucleotide polymorphism array, gene duplication, *PLP1*, multiplex ligation-dependent probe amplification, prenatal diagnosis

## Abstract

A family with a history of Pelizaeus-Merzbacher disease (PMD) received prenatal diagnosis of *PLP1* gene duplication in a fetus using a single nucleotide polymorphism (SNP) array. A 27-year-old pregnant woman was referred for genetic counseling due to her four-year-old son being diagnosed with a suspected classic type of PMD. Amniocentesis was performed at 18 and 3/7 weeks of gestation, and the SNP array was carried out on DNA from the mother, her affected son, and fetus, then further confirmed by multiplex ligation-dependent probe amplification (MLPA). Cytogenetic analysis of the fetus showed 46,XY. SNP array analysis revealed that the male fetus did not carry *PLP1* gene duplication but the affected boy did, and the mother was a carrier for the duplication of the *PLP1* gene. All SNP array results were further confirmed by MLPA. SNP array and MLPA analyses of peripheral blood verified the nonduplication of the *PLP1* gene in the infant after birth. At present, the child (without *PLP1* duplication) is developing normally. This study preliminarily suggests that SNP array is a sensitive and accurate technology for identifying *PLP1* duplication and is feasible for reliable diagnosis, including for the prenatal diagnosis of PMD resulting from *PLP1* duplication.

## INTRODUCTION

Pelizaeus-Merzbacher disease (PMD; MIM 312080) is a rare X-linked recessive demyelinating central nervous system (CNS) disorder [1]. The gene that causes PMD is *PLP1*, which is located on chromosome Xq22.2. *PLP1* consists of seven exons that encode two splicing isoforms (PLP1 and DM20). Affected male patients’ mothers are often carriers of *PLP1* variations and are often asymptomatic, thus, they are at a 50% risk of having male children with PMD. Currently, there is no definitive treatment for PMD. Therefore**,** accurate prenatal genetic diagnoses are necessary for high-risk couples.

Approximately 60-70% of PMD cases arise from complete genomic duplication of the *PLP1* gene [2], and fewer result from *PLP1* deletion [3] and point mutations [4]. Clinical symptoms and signs of PMD include spastic paraplegia, nystagmus, cerebellar ataxia, psychomotor developmental delay (DD), and dystonia. Brain magnetic resonance imaging (MRI) of patients with PMD has revealed normal myelin and oligodendrocyte reduction in the brain, leading to myelination delay [5]. Typically, patients with *PLP1* duplication manifest with the classic type of PMD, whereas patients with point mutations commonly present with the more severe type of PMD [2].

The phenotypes of patients carrying *PLP1* duplication vary greatly, ranging from mild to severe PMD, but most present with the classic type of PMD. Genetic testing of PMD is vital for counseling and family planning for a PMD family. Duplications of the *PLP1* gene have been identified by array comparative genomic hybridization (aCGH) [6], fluorescent *in situ* hybridization (FISH) [7], multiplex ligation-dependent probe amplification (MLPA) [8], quantitative PCR [9], multiplex amplification and probe hybridization (MAPH) [10, 11], and real-time PCR [12]. Furthermore, MLPA is a reliable method and has marked advantages over other technologies, such as aCGH, FISH, and MAPH [8, 13]. Chromosomal microarray analysis (CMA) such as aCGH [7] do not only accurately detect genomic copy number variations (CNVs) but can identify duplication of the *PLP1* gene [14].

Similarly, prenatal diagnosis of fetal chromosome abnormalities as well as *PLP1* duplication are effectively identified through invasive prenatal diagnosis technologies, such as chorionic villus sampling, amniocentesis, or cordocentesis, depending on the gestational age and other circumstances [15]. Here, we present one PMD family with *PLP1* duplication and performed prenatal diagnosis of the fetus using a single nucleotide polymorphism (SNP) array. Further MLPA analysis on *PLP1* duplication was performed to validate the SNP array results. The pregnant woman was at risk of being a carrier of *PLP1* duplication due to her son having suspected PMD, and she requested prenatal diagnosis of the pregnancy. The proband, the son’s pregnant mother, was tested first, and amniocytes were cultured from the fetus using an SNP array, before validation by MLPA. Herein, we preliminarily suggest that SNP array is a reliable alternative prenatal diagnosis technology for detecting *PLP1* duplication.

## RESULTS

### Brain magnetic resonance imaging of the proband

**Figure 1:**
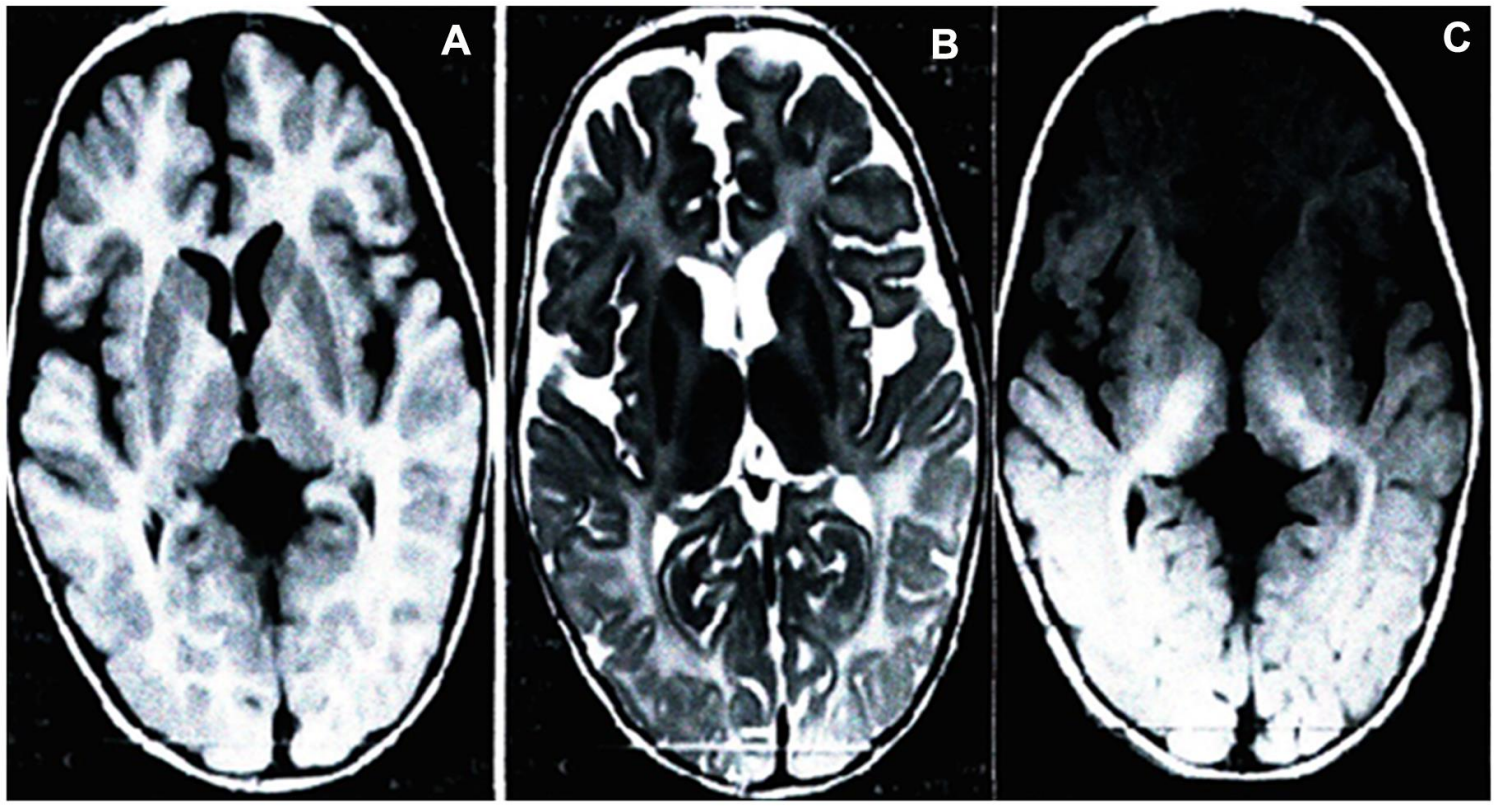
**Brain MRI of the proband.** Scans showed significantly development delay of white matter, poor myelin formation, close to the level of myelin development in neonates, and reduced volume of white matter throughout the brain. Axial T1-weighted (**A**), T2-weighted (**B**) and FLAIR (**C**).

Brain MRI scans of the proband (the son of the pregnant woman) displays aberrant white matter and no normal myelin signal in the supratentorial structures, with homogeneously high signal intensities in the white matter on T1-weighted (Figure 1A), subcortical new fiber on the T2-weighted (Figure 1B) and fluid attenuated inversion recovery (FLAIR) images (Figure 1C). The cerebellar white matter lacking the normal dark myelin signal on T2-weighted images (Figure 1B), showing abnormal myelination of white matter in the brain. The brain MRI findings of the proband were consistent with PMD.

### Cytogenetic analysis

**Figure 2:**
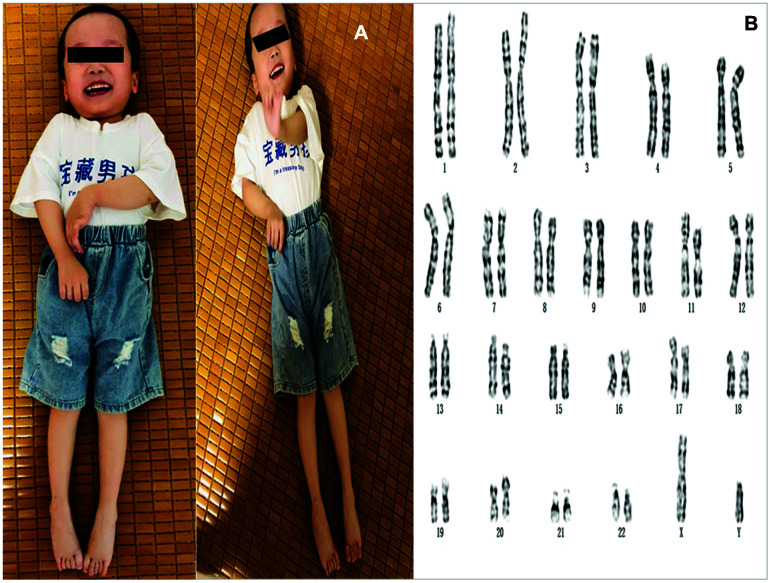
(**A**) The photos of the proband. (**B**) Karyotyping analysis of the fetus. Chromosomal analysis on cultured amniocytes rvealed a normal male fetus (46,XY).

Cytogenetic analysis of the cultured amniocytes revealed a normal male karyotype (46,XY) (Figure 2).

### SNP array analysis

**Figure 3:**
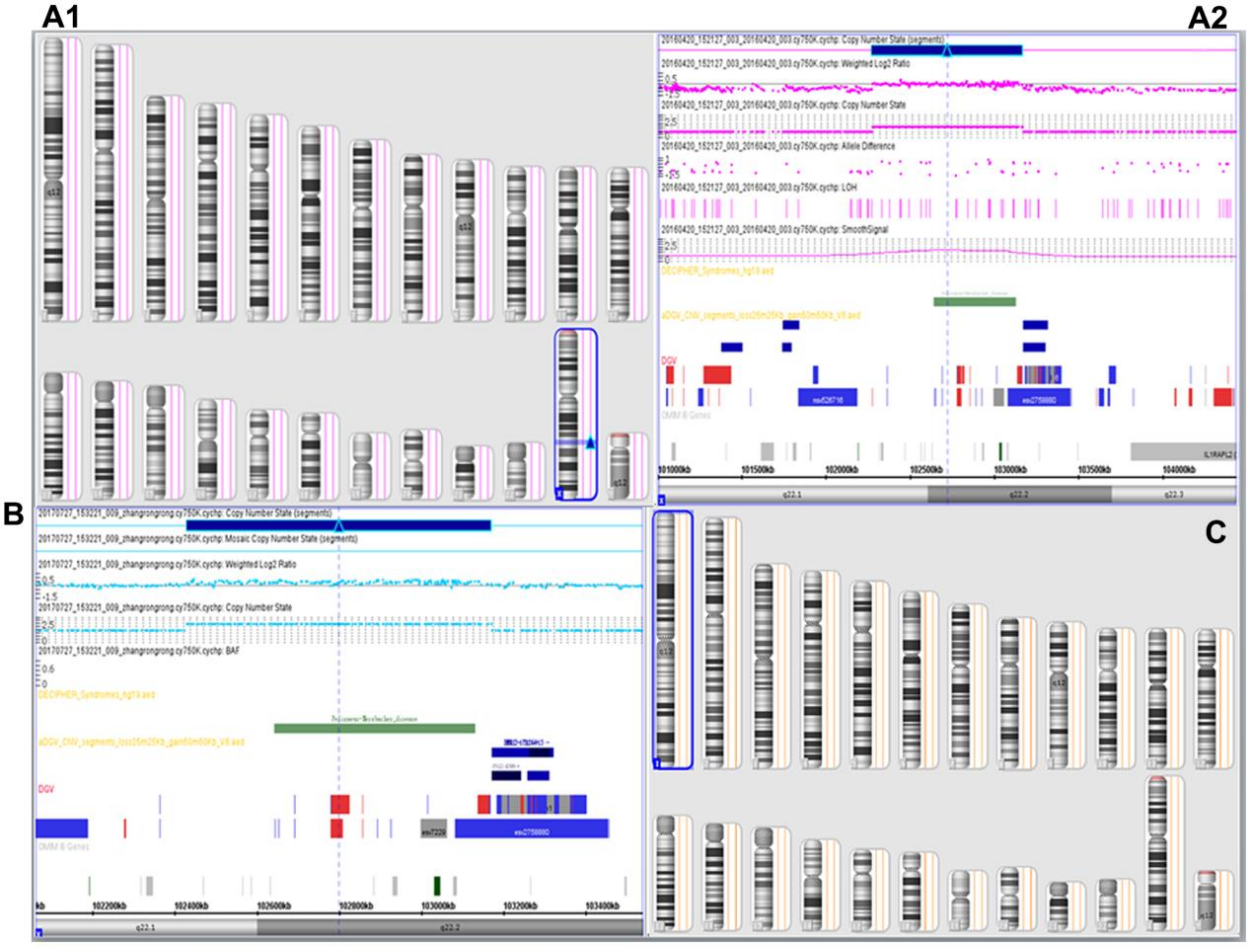
**SNP array results.** SNP-array analysis showed the proband (**A1**, **A2**) and his mother (**B**) to be 46,XY.arr[GRCh37]Xq22.1q22.2 (102,429,064-103,168,721)x2 and arr[GRCh37] Xq22.1q22.2(102,429,064-103,168,721)x3, respectively, with a duplicated 740 Kb region (102,429,064-103,168,721) leading to partial disomy of Xq22.1q22.2, which encompassing *PLP1* gene, the SNP array results of the boy indicated a diagnosis of PMD. The pregnant mother was proved to be a female carrier of *PLP1* duplication, and the male fetus revealed no such duplication (arr[GRCh37] (1-22)x2, (XY)x1) (**C**, Table 1).

The SNP array analysis of genomic DNA from the pregnant woman and her four-year-old son revealed both the proband and his pregnant mother had a 740 Kb microduplication in the Xq22.1q22.2 chromosomal region (chrX: 102,429,064-103,168,721, GRCh37), containing eight OMIM genes: *BEX4* (300692), *BEX2* (300691), *TCEAL7* (300771), *BEX3* (300361), *TCEAL1* (300237), *MORF4L2* (300409), *RAB9B* (300285), and *PLP1* (300401), including *PLP1* gene duplication (Figure 3). In the pedigree, SNP array analysis showed a 740 Kb duplication at chromosome Xq22.1q22.2 (46,XX.arr[GRCh37] Xq22.1q22.2(102,429,064_103,168,721)x3) in the pregnant woman (B) and her son (the proband) (46,XY.arr[GRCh37]Xq22.1q22.2(102,429,064_103,168,721)x2) (A) (Table 1). Whereas, the male fetus revealed no such duplication (C).

**Table 1:** *PLP1* gene duplications subjects identified using SNP array.

**Cases**	**Sex**	**SNP array**	**Duplicated OMIM genes**
The proband	Male	740 Kb duplication at Xq22.1q22.2	*BEX4, BEX2, TCEAL7, BEX3, TCEAL1,*
		(46,XY.arr[GRCh37]Xq22.1q22.2(102,429,064_103,168,721)x2)	*MORF4L2, RAB9B, PLP1*
The pregnant woman	Female	740 Kb duplication at Xq22.1q22.2	*BEX4, BEX2, TCEAL7, BEX3, TCEAL1,*
		(arr[GRCh37] Xq22.1q22.2(102,429,064_103,168,721)x3)	*MORF4L2, RAB9B, PLP1*
The fetus	Male	(arr[GRCh37] (1-22)x2, (XY)x1)	no

### MLPA results

**Figure 4:**
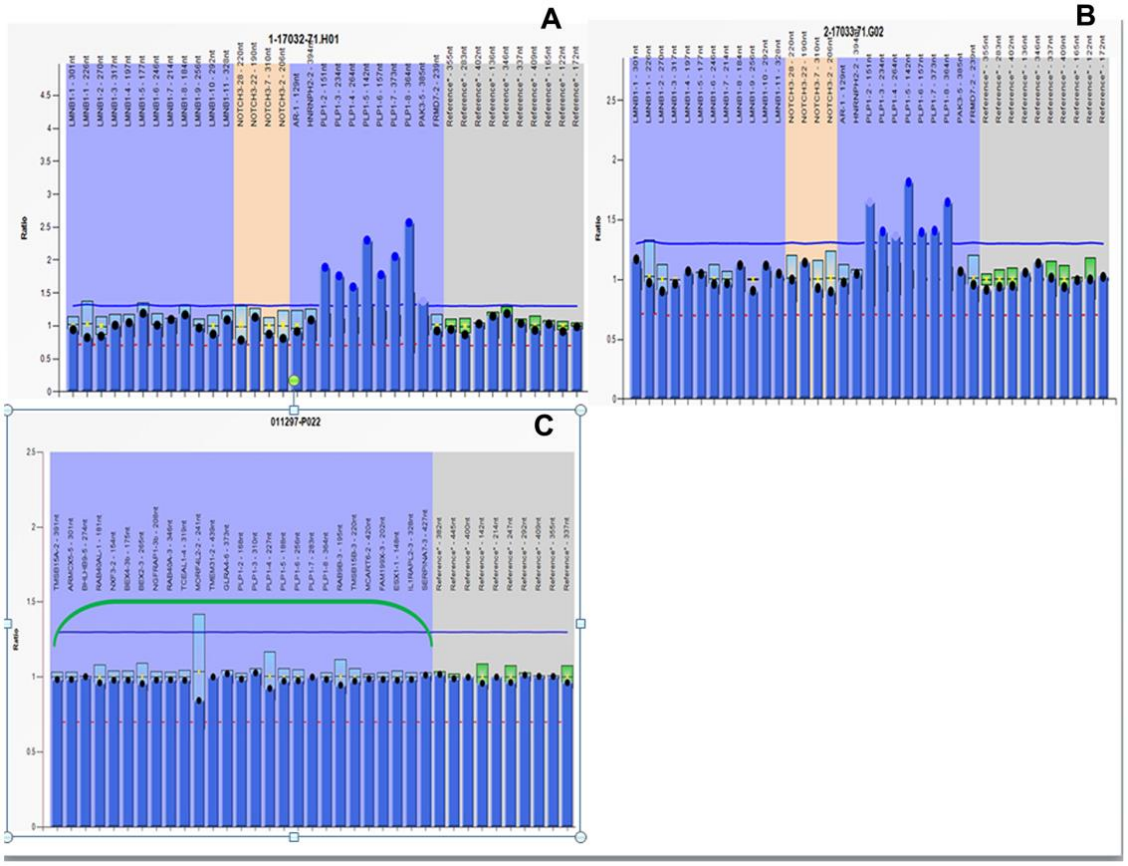
**MLPA results.** All peaks corresponding to the 7 exons of the *PLP1* gene in the proband and his mother are higher than peaks in female control due to *PLP1* gene duplication. The relative copy numbers for each *PLP1* exon were shown. Fluorescence signal intensity between 0.7 and 1.25 is generally considered normal result. (**A**) MLPA results of the proband. (**B**) MLPA results of the pregnant mother. (**C**) MLPA results of the fetus.

MLPA analysis of the pregnant woman revealed partial duplication of Xq22.1q22.2 and duplication of the *PLP1* gene, confirming her status as a *PLP1* duplication female carrier (Figure 4A). *PLP1* duplication was also found in her four-year-old son (Figure 4B), validating the SNP array diagnosis results. MLPA analysis of cultured amniocytes revealed that the male fetus did not have *PLP1* duplication (Figure 4C), also confirming the original SNP array analysis. Taken together, these findings indicate that the proband could be diagnosed with PMD caused by the duplicated *PLP1* gene, inherited from his unaffected carrier mother.

### Follow-up studies

The pregnant woman gave birth to a phenotypically normal male child, whose early development has been normal. At two months of age, he did not show any signs of PMD, whereas at this age, his affected brother presented with horizontal nystagmus. Repeat SNP array analysis of peripheral blood revealed the same result of *PLP1* nonduplication (data not shown), and verified the prenatal genetic testing results. This was also confirmed in a postnatal physical examination and subsequently at three months of age. At present, he is an 11-month-old child with normal developmental milestones and no signs of PMD.

## DISCUSSION

PMD is a congenital hypomyelination disorder. The gene causing PMD is *PLP1* on chromosome Xq22.2. *PLP1* consists of seven exons that encode two splicing isoforms (PLP1 and DM20). PLP1 is mainly expressed and accounts for more than 50% of the protein in oligodendrocytes [16]. *PLP1* gene duplication is the most common cause of PMD, at 100 Kb to ~5 Mb in size [14, 17]. The size of the duplicated region is variable due to the *PLP1* complex genomic region including several flanking low-copy repeats [18]. Therefore, PMD is considered an X-linked recessive chromosomal disorder [19]. As the clinical severity spectrum overlaps despite the tendencies in the genotype-phenotype correlations, it is not reliable for predicting the phenotypes of a patient with a specific genotype based on clinical features alone [20]. Thus, accurate molecular diagnosis of PMD is vital.

PMD is clinically heterogeneous and ranges from severe connatal PMD to mild PMD/spastic paraplegia, but most patients have the classic form of PMD [21]. Two thirds of patients with PMD have *PLP1* duplications and show the classic type of PMD, and three or more copies of the *PLP1* cause more severe PMD [8]. *PLP1* duplications are commonly generated by intrachromosomal sister chromatid exchange during meiosis [22]. Wolf et al. [8] demonstrated that the level of *PLP1* expression correlates with disease severity. However, few cases of *PLP1* duplication presenting severe phenotypes have been reported [14]. There appears to be no relationship between the size of the duplicated region and phenotypic severity. Increased gene dosage leads to the overexpression of the *PLP1* gene [23], which disrupts the assembly of membrane rafts and causes accumulation of PLP1 with cholesterol and lipids in the late endosomal/lysosomal compartments, leading to mature oligodendrocyte apoptotic cell death and the developmental arrest of immature oligodendrocytes [24].

When we detected *PLP1* duplications in the probands, testing of their mothers was often necessary. In addition, when mothers carry *PLP1* duplication, accurate prenatal genetic testing is mandatory for future pregnancies. FISH, MLPA, aCGH, and droplet-digital polymerase chain reaction (ddPCR) have been used to detect *PLP1* duplications in patients with PMD and their carrier mothers [25] (Table 2 [14, 15, 25–28]).

**Table 2:** Main technologies for testing the duplications of PLP1 gene.

**Technology**	**Advantage**	**Disadvantage**	**Ref.**
FISH	Chromosomal balanced rearrangement in metaphase and duplications in interphase can be detected	Even in interphase, small duplications could not be diagnosed. it is sometimes difficult to detect duplications on the same chromosome, the interpretation of results is subjective.	[15, 27]
MLPA	small duplications/deletions can be accurately detected, can detect small duplication	Cannot detect signal intensity accurately cannot detect the size of the duplication, need extend beyond neighboring genes in case of large size of duplication	[28]
aCGH	Both the duplicated region and size can be detected. can detect small duplication.	Chromosome balanced rearrangement cannot be diagnosed cannot detect LOH and low level mosaicism	[14]
ddPCR	Cannot detect signal intensity triplicate experiments are no longer necessary, can rapidly detect PLP1 duplication with very small amounts of DNA, needs only 20 ng, more rapidly (only 6 hours) required assay time		[26]
qPCR	Easy to diagnosis duplication/deletion maybe ambiguous	In cases with, sometimes the duplication diagnosis result	[29]
SNP array	A more efficient methods than aCGH, can detect not only duplications, but also identify extent of duplications. Mosaicism cannot be diagnosed can detect small duplication.	Chromosome balanced rearrangement and low proportion cannot be used for screening.	

aCGH is a feasible alternative technology for the detection of *PLP1* duplication, and its advantages over karyotyping and FISH in prenatal diagnosis have been reviewed [7]. For aCGH technology, it is impossible to detect chromosomal abnormalities with normal copy numbers, such as chromosomal balanced rearrangement, uniparental disomy (UPD), and loss of heterozygosity (LOH). Furthermore, it can miss small *PLP1* duplications/deletions and mosaicism, with the wide implementation of another type of CMA analysis, SNP array in prenatal diagnosis. SNP array is an accurate and rapid technology for detecting CNVs with advantages over karyotyping, aCGH, and FISH. SNP array chip contains many high-density SNP probes and can not only detect CNVs, but also identify UPD, LOH, and low-level mosaicism. In addition, the SNP array assay does not require the reference of genomic DNA of the normal control population, thus, avoiding the interaction between two fluorescent dyes. In addition, SNP arrays have a higher resolution than aCGH, thus, it can identify microduplications/microdeletions over dozens of Kb, providing more detailed and comprehensive information. Since PMD is mostly caused by submicroscopic chromosomal anomalies [19] due to the duplication of the *PLP1* gene [7], SNP arrays are not only informative in characterizing the size of the genomic CNVs but may also be useful in determining the *PLP1* copy number. It takes approximately one-half to one full day to perform an SNP array. Therefore, the SNP array is more efficient than aCGH.

Genetic testing of PMD is important for affected patients’ families because female carriers with *PLP1* duplication will have a male fetus with 50% risk of inherited PMD and a female fetus with 50% risk of carrying *PLP1* duplication in any future pregnancies. Because PMD is a chromosomal microduplication/microdeletion disorder [19], herein, we report an SNP array method for diagnosis and prenatal diagnosis of PMD resulting from *PLP1* duplication, the SNP array results of *PLP1* duplication were further validated by MLPA. We preliminarily suggest that SNP array technology is reliable and accurate for detecting *PLP1* duplication as well as CNVs.

PMD can be diagnosed based on clinical manifestations and brain MRI results. Barkovich [5] suggested that brain MRI of patients with PMD reveal normal myelin and oligodendrocyte reduction, causing delayed myelination. Combining clinical manifestations, brain MRIs (Figure 1), and molecular diagnosis results, the four-year-old boy was diagnosed with classic type of PMD, and his mother, who was carrying a fetus at the time of diagnosis, underwent genetic counseling and requested amniocentesis at 18 and 3/7 weeks of gestation. Prenatal karyotyping analysis of amniocytes showed 46,XY (Figure 2). First, an SNP array was attempted to detect *PLP1* duplication in the peripheral blood from the four-year-old boy (the proband) (Figure 3A) and his mother. (Figure 3B). SNP array analysis revealed that both the proband and his mother were carrying *PLP1* duplications, thus, confirming the diagnosis of PMD and *PLP1* duplication carrier, respectively. Second, the SNP array results were further verified by MLPA (Figure 4A, 4B).

PMD is a submicroscopic chromosomal disorder [19] often caused by duplications of the *PLP1* gene [7]. Therefore, the SNP array is also feasible in detecting *PLP1* duplication, in theory. In addition, MLPA can diagnose PMD resulting from *PLP1* duplications [8] and is an accepted technology for detecting *PLP1* duplications or deletions in most testing companies and medical institutions. Finally, an SNP array was performed on cultured amniocytes from the male fetus. Fortunately, the male fetus did not carry *PLP1* duplication (Figure 3C), and further MLPA analysis yielded the same *PLP1* nonduplication result (Figure 4C). Therefore, we predicted a healthy male baby without PMD to be born in due time. SNP array and MLPA analyses were also performed on DNA extracted from the peripheral blood of the infant after birth and the same results were seen (data not shown), confirming the prenatal diagnosis results.

The limitation of our study is that the SNP array does not identify *PLP1* point mutation, low proportion mosaicism (< 30%), or balanced rearrangement, such as intragenic genomic rearrangement of the *PLP1* gene resulting in PMD, and SNP array characterized *PLP1* duplications require a large population study in future.

In this case study, *PLP1* duplications in patients with PMD and female carriers were accurately diagnosed for the first time, to our knowledge, by SNP array. Therefore, SNP arrays can be used in the same manner as MLPA and aCGH. We preliminarily suggest that SNP array is a rapid and accurate method for diagnosing *PLP1* duplication, even in prenatal diagnosis.

## MATERIALS AND METHODS

### Study subjects

The pedigree (pregnant woman) was a 27-year-old G2P1 pregnant woman at 18 and 3/7 weeks of gestation. She was admitted to our hospital due to adverse reproductive history of suspected classic type of PMD. After further continuing to question the family history, there were three affected male members of her pedigree with suspected PMD. The proband was the pregnant woman's four-year-old son, the boy is the first child of unrelated, healthy, young parents. He was born at 39 and 2/7 weeks of gestation, pregnancy and delivery were uneventful. The male baby weighing 2680 g at birth and with an Apgar score of 7 showed first sign of a CNS insults, he exhibited DD, ataxia, language impairment, mental retardation, seizures, and progressive difficulty in sitting and walking: He presented early onset horizontal nystagmus three weeks after birth, hypotonia, along with DD was noticed during the second month of life, his psychomotor development was severely retarded at five months of age, and he was unable to hold his head up until seven-eight months because of hypotonia, and he can't sit alone at nine months, and never walk alone due to increased tendon reflexes up to now, and presented almost no active movement such as turn around or sit alone, he can only speak monosyllabic words at three years old, very little progress has happened since then.

The 27-year-old mother conceived with a second child, after obtaining informed consent, a transabdominal amniocentesis under ultrasonic guidance was performed at the 18 and 3/7 weeks of gestation, the prenatal diagnosis on cytogenetic analysis and *PLP1* duplication status of the fetus using SNP array is performed. The pregnant woman had no complications of pregnancy. Level III Doppler ultrasound revealed no apparent fetal abnormality at 23 weeks of gestation.

### Sample collection

An amniotic fluid sample was taken by amniocentesis at 18^+3^ weeks and 30 mL of amniotic fluid was extracted for chromosome karyotype analysis, SNP array analysis, and MLPA. Peripheral blood was sampled from the pregnant woman and her son, while the other two maternal male patients have possibly been affected by PMD, and denied further genetic test, their death occurred at the age of 8 years and 10 years, respectively.

### Isolation of genomic DNA

Genomic DNA from the boy and his mother (the pregnant woman) was obtained from 2-4 mL of peripheral blood after informed consent. Fetal genomic DNA was isolated from cultured amniocytes and ruled out for maternal cell contamination using microsatellite DNA linkage analysis. DNA was extracted using the QIAamp® DNA Blood Mini Kit (QIAGEN), and the concentration and purity of genomic DNA was measured by a NanoDrop micro-volume UV-Vis spectrophotometer (Thermo Fisher Scientific).

### Brain magnetic resonance imaging

Verio 3.0 T superconducting MAGNETIC resonance scanner (German Siemens) is adopted by scan score for gradient recalledecho (GRE), fluid attenuated inversion recovery (FLAIR), diffusion weighted imaging (DWI), and susceptibility weighted imaging (SWI) positive results according to the number, size, and location of lesions.

### Cell culture and cytogenetic analysis

Amniotic fluid cells from the fetus were routinely cultured and subjected to G-band karyotyping (~500 bands). The cytogenetic findings were described according to the International System for Human Cytogenetic Nomenclature 2016 (ISCN 2016).

### SNP array analysis

Genomic DNA of the boy, the pregnant woman and the fetus was digested, amplified, purified, fragmented, marked with signals, hybridized on the Affymetrix CytoScan 750K array (Affymetrix), and washed, and images were acquired. The data obtained was processed with the software Chromosome Analysis Suite (Affymetrix). The reporting threshold was set at ≥200 kb for loss and ≥400 kb gain in the study.

### MLPA analysis

Lymphocytes were harvested from peripheral blood of the pregnant woman and her four-year-old son for MLPA analysis described previously [29]. Cultured amniocytes and lymphocytes from peripheral blood of the infant were harvested post delivery. MLPA was performed to validate our SNP array results. A MLPA kit (SALSA MLPA KIT P022; MRC Holland, Amsterdam, Netherlands) was used to screen all exons of the *PLP1* gene. The probe mix included 32 probes, of which seven were for each of the *PLP1* exons, eight were from different regions of the X chromosome, one was from the Y chromosome and the remainders were autosomal controls. Details of probe sequences, gene loci and chromosome locations can be found at http://www.mrc-holland.com. The reactions were carried out in a thermal cycler according to the manufacturer’s instructions, the reaction products were detected with an ABI 3100 Genetic Analyzer (Applied Biosystems). We used GeneScan and Genotyper software to size the PCR products and to obtain peak areas, the data was analyzed using Coffalyser software. PCR and Sanger sequencing were performed to verify the deletion of corresponding exons. Data analysis was performed with GeneMarker® software (SoftGenetics LLC, State College, PA 16803, U.S.A.). Expected *PLP1* values for males with *PLP1* duplication, female carrier of *PLP1* duplication, non-PMD females, and non-PMD males are 2, 1.5, 1, and 1, respectively.

### Ethical statement

This study was reviewed and approved by the Ethics Review Committee of Fujian Provincial Maternity and Children’s Hospital (approval no: 2007-0112). Signed informed consent was obtained from all participants following a detailed description of the purpose of the study. All experiments were performed in accordance with relevant guidelines and regulations.
